# Efficacy and safety of therapeutic exercise for primary dysmenorrhea: a systematic review and meta-analysis

**DOI:** 10.3389/fmed.2025.1540557

**Published:** 2025-02-26

**Authors:** Yi Xiang, Qinhai Li, Zengao Lu, Zemin Yu, Guanglin Ma, Siqi Liu, Yingkui Li

**Affiliations:** ^1^China Wushu School, Beijing Sport University, Beijing, China; ^2^School of Sports and Health, Shanghai University of International Business and Economics, Shanghai, China; ^3^School of Physical Education, Hunan Normal University, Changsha, China

**Keywords:** primary dysmenorrhea, exercise, pain, visual analog scale, women’s health

## Abstract

**Objective:**

A growing number of research papers have looked at the influence of exercise on pain severity in people with primary dysmenorrhea, but the findings have been inconsistent. The purpose of this research was to thoroughly evaluate the impact of exercise on pain severity in individuals with primary dysmenorrhea and to find the best exercise regimen for these patients.

**Methods:**

All randomized controlled trials (RCTs) of exercise for patients with primary dysmenorrhea were searched in the Embase, PubMed, Cochrane, Web of Science, EBSCO, and CINAHL databases. The search time limit was set from the establishment of each database to 28 January 2025. Control groups included those receiving usual care, only providing health education, or no intervention at all. The outcome measure was pain intensity. The effect size was aggregated using the mean difference (MD) and 95% confidence interval (CI). The methodological quality of the included studies was evaluated using the Cochrane risk-of-bias tool. Stata 15 software was used for meta-analysis, sensitivity analysis, and assessment of potential publication bias. R 4.4.1 software was used for network meta-analysis, area under curve ranking (SUCRA), league plot, and meta-regression.

**Results:**

The analysis included a total of 29 studies that satisfied the criteria. Exercise decreased the visual analog scale (VAS, WMD = −2.62, 95% CI [−3.29, −1.95], *p* < 0.001) score in individuals with primary dysmenorrhea. Strength training (WMD = −1.76, 95% CI [−2.03, −1.48], *p* < 0.001), exercise duration of ≥8 weeks (WMD = −1.77, 95% CI [−1.87, −1.66], *p* < 0.001), frequency of >3 times per week (WMD = −1.60, 95% CI [−1.75, −1.45], *p* < 0.001), session length of >30 min (WMD = −2.20, 95% CI [−2.38, −2.02], *p* < 0.001), and a total of ≥90 min per week (WMD = −2.04, 95% CI [−2.19, −1.89], *p* < 0.001) showed superior efficacy in enhancing VAS (subgroup analyses).

**Conclusion:**

Engaging in physical activity may reduce the intensity of discomfort encountered by individuals afflicted with primary dysmenorrhea, with strength training potentially emerging as the most efficacious alternative. The meta-analysis presents evidence that supports clinicians’ advice to patients with primary dysmenorrhea, recommending that they exercise for a minimum of 8 weeks, with sessions occurring more than three times weekly and lasting longer than 30 min each. The goal is to achieve a minimum weekly total of 90 min by increasing the frequency of workouts.

**Systematic review registration:**

https://www.crd.york.ac.uk/prospero/display_record.php?ID=CRD42024581879, CRD42024581879.

## 1 Introduction

Dysmenorrhea represents a common gynecological symptom encountered by numerous women of reproductive age during their menstrual cycle (1), and its prevalence varies significantly across the world, ranging from 24 to 92% (2, 71). Despite its considerable occurrence, the diagnostic process frequently falls short, leading to numerous patients refraining from pursuing medical assistance (3). In addition, dysmenorrhea, as a common gynecological disorder, poses a significant and non-negligible negative impact on women’s quality of life, ability to perform daily activities, occupational performance, and academic achievement (1, 4), and it may also cause a serious social and economic burden. For example, in the United States, dysmenorrhea triggers an estimated direct economic loss of approximately $600 million per year, which equates to approximately 2 billion work hours not being utilized effectively each year (5).

The ICD-10 classification delineates dysmenorrhea into two distinct categories: primary dysmenorrhea and secondary dysmenorrhea (6). Primary dysmenorrhea typically manifests within 6–12 months following menarche (7). Primary dysmenorrhea is not associated with organic lesions but is primarily attributed to uterine smooth muscle spasms and vasoconstriction, which are induced by elevated prostaglandin levels (8). Secondary dysmenorrhea is linked to pelvic organ pathologies such as endometriosis, uterine fibroids, or adenomyosis. Research indicated that the intensity of primary dysmenorrhea pain peaks at the onset of menstruation and may be accompanied by symptoms including diarrhea, nausea, vomiting, and headache (7, 9). Moreover, the prevalence of dysmenorrhea varies significantly in different regions. For example, in European countries, the average prevalence ranges from 45 to 97%, with Bulgaria reporting the lowest rate (8.8%) and Finland reporting the highest rate (94%) (10). In Indonesia, the overall prevalence of dysmenorrhea is 64.25%, of which 54.89% is classified as primary dysmenorrhea (10).

At present, certain interventions, including non-steroidal anti-inflammatory drugs (NSAIDs) and oral contraceptives, have shown efficacy in the management of primary dysmenorrhea. Nevertheless, the administration of non-steroidal anti-inflammatory medications (NSAIDs) can entail gastrointestinal disturbances such as irritation or ulcer development (even when accompanied by stomach-protecting drugs) (11), and their prolonged application may give rise to issues concerning the cardiovascular, liver, and kidney systems. Similarly, the utilization of oral contraceptives might elevate the occurrence of bleeding incidents, contribute to weight gain, or heighten the patients’ vulnerability to venous thromboembolic events (12). All of this highlights the need for an alternative to conservative therapy. Therefore, an increasing number of studies are focusing on non-pharmacological alternative therapies, such as physical exercise. Physical exercise is not only cost-effective and low-risk with no side effects (13, 14) but also improves mental health and quality of life. For instance, activities such as aerobic exercise and yoga have been proven to effectively reduce the pain intensity and discomfort associated with dysmenorrhea (15–19).

Dysmenorrhea not only severely affects women’s physical and mental health but also has a profound impact on society and the economy. One study showed that the average number of absence days for students with severe dysmenorrhea is 3.46 days (20). Another study indicated that the rate of absenteeism due to dysmenorrhea among young women was between 34 and 50% (21). This not only impacts an individual’s academic and professional development but also increases medical costs and social burden. Despite the various alternative treatments that have been proposed, there remains controversy over which method is most effective. Furthermore, previous studies failed to concentrate on specific types of intervention, frequency, session length, and weekly time, resulting in considerable disparities across therapies. As a result, we did a thorough systematic review and meta-analysis of randomized controlled trials (RCTs) to investigate the effects of exercise on pain intensity in patients with primary dysmenorrhea and to find the best exercise prescription.

## 2 Methods

The research was conducted in accordance with the Cochrane Selection Manual (22) and the Preferred Reporting Items (23) for Systematic Reviews and Meta-analyses guidelines (71). The protocol has been duly registered with PROSPERO (CRD42024581879).

### 2.1 Search strategy

Two independent researchers (YX and QL) screened and selected participants. In times of dispute, a third reviewer (YL) was consulted to encourage cooperation and reach a consensus. To compile all pertinent RCTs, we conducted a comprehensive literature review across six esteemed databases: Embase, PubMed, Cochrane, Web of Science, EBSCO, and CINAHL. The search time limit was set from the inspection of each database to 28 January 2025. The search terms used included: (“Exercise”[Mesh] OR “physical Exercise” OR “physical activity” OR “aerobic exercise” OR “isometric exercise” OR “acute exercise” OR “exercise training”) AND (“Dysmenorrhea”[Mesh] OR “painful menstruation” OR “menstrual pain” OR “primary dysmenorrhea” OR “menstrual cramps” OR “painful periods”) AND (“Randomized Controlled Trial”[Mesh] OR “controlled clinical trial” OR “randomized” OR “randomised” OR “controlled” OR “trial” OR “random” OR “placebo” OR “groups”).

### 2.2 Eligibility criteria

The inclusion criteria in this research were as follows: Criteria for qualifying research requirements (1): A randomized controlled trial design was used (2); Both intervention and control groups were incorporated (3); Participants experiencing primary dysmenorrhea were involved. The intervention group only received exercise intervention, and the control group did not receive exercise intervention (4); the VAS served as an essential indicator for evaluating the outcome. The criteria for exclusion encompassed the following factors (1): publications in languages other than English (2); review articles and conference proceedings (3); studies that utilized animal models (4); exercise interventions designated for the control group (5); studies that included women with abnormal menstrual cycles (6); studies that included women diagnosed with gynecological diseases, surgery, or serious diseases (7); studies that included women using intracavitary or oral contraceptives.

### 2.3 Data extraction

Data extraction was performed independently by two authors (YX and QL). The data extraction covered the following topics: (a) study characteristics, such as the first author’s surname, year of publication, country of publication, and sample size; (b) intervention specifics, such as the type, frequency, duration, and length of each session; (c) participant demographics, especially age; and (d) treatment outcomes, such as the mean and standard deviation (SD) values indicating changes in pain intensity after the intervention.

### 2.4 Methodological quality assessment

The quality assessment adhered to the guidelines specified in the Cochrane Intervention System Evaluation Manual. The assessment criteria encompassed randomization methods, allocation concealment, blinding, completeness of outcome data, selective reporting of results, and other sources of bias. The degree of risk was classified as low when criteria were met, high when they were not met, and moderate if not defined in the article. Two independent researchers completed the quality evaluation. Any inconsistencies were handled by consulting a third researcher to establish an agreement.

### 2.5 Statistical analysis

The meta-analysis was performed utilizing Stata 15 software; however, it faced limitations owing to the clinical heterogeneity present in the included studies. The I^2^ statistic functioned as a metric for quantifying heterogeneity, with values indicating low (25%), intermediate (50%), and high (75%) levels. A fixed effects model was adopted if *I*^2^ < 50% or *p* > 0.1, suggesting no statistical heterogeneity between groups. If these criteria were not satisfied, a random effects model was utilized. The data obtained were sourced from the visual analog scale (VAS) and analyzed based on the mean difference, accompanied by a 95% confidence interval. Forest plots were developed to visualize the overall advantageous impact of the intervention on pain mitigation, whereas funnel plots were constructed to assess potential publication bias.

By using network meta-analysis, we aimed to systematically evaluate and rank the average disparities in pain relief among diverse exercise regimens for individuals with primary dysmenorrhea. R 4.4.1 software was used to choose a fixed or random effects model based on the clinical heterogeneity of the included studies, and the study outcome indicators were networked using grouping instructions for network meta-analysis. Data processing, area under the curve ranking (SUCRA), league plot, and meta-regression were conducted sequentially. The strengths and weaknesses of the therapies were evaluated according to the SUCRA value. A SUCRA of 1 indicates complete therapeutic success, while a SUCRA of 0 denotes ineffectiveness.

We used the following criteria for subgroup analyses: minutes per intervention (≤30 min and > 30 min), minutes per week (<90 min and ≥ 90 min), frequency (≤3 times per week and > 3 times per week), intervention length (<8 weeks and ≥ 8 weeks), and type of intervention (aerobic exercise, mind–body therapy, stretching exercise, strength training, relaxation exercise, and multicomponent training). Forest plots were produced utilizing Stata 15 software, succeeded by sensitivity analysis and funnel plots. The R 4.4.1 application generated the area under the curve ranking (SUCRA), league plot, and meta-regression analysis. The results with *p* < 0.05 were considered statistically significant.

## 3 Results

### 3.1 Study selection

**Figure 1:**
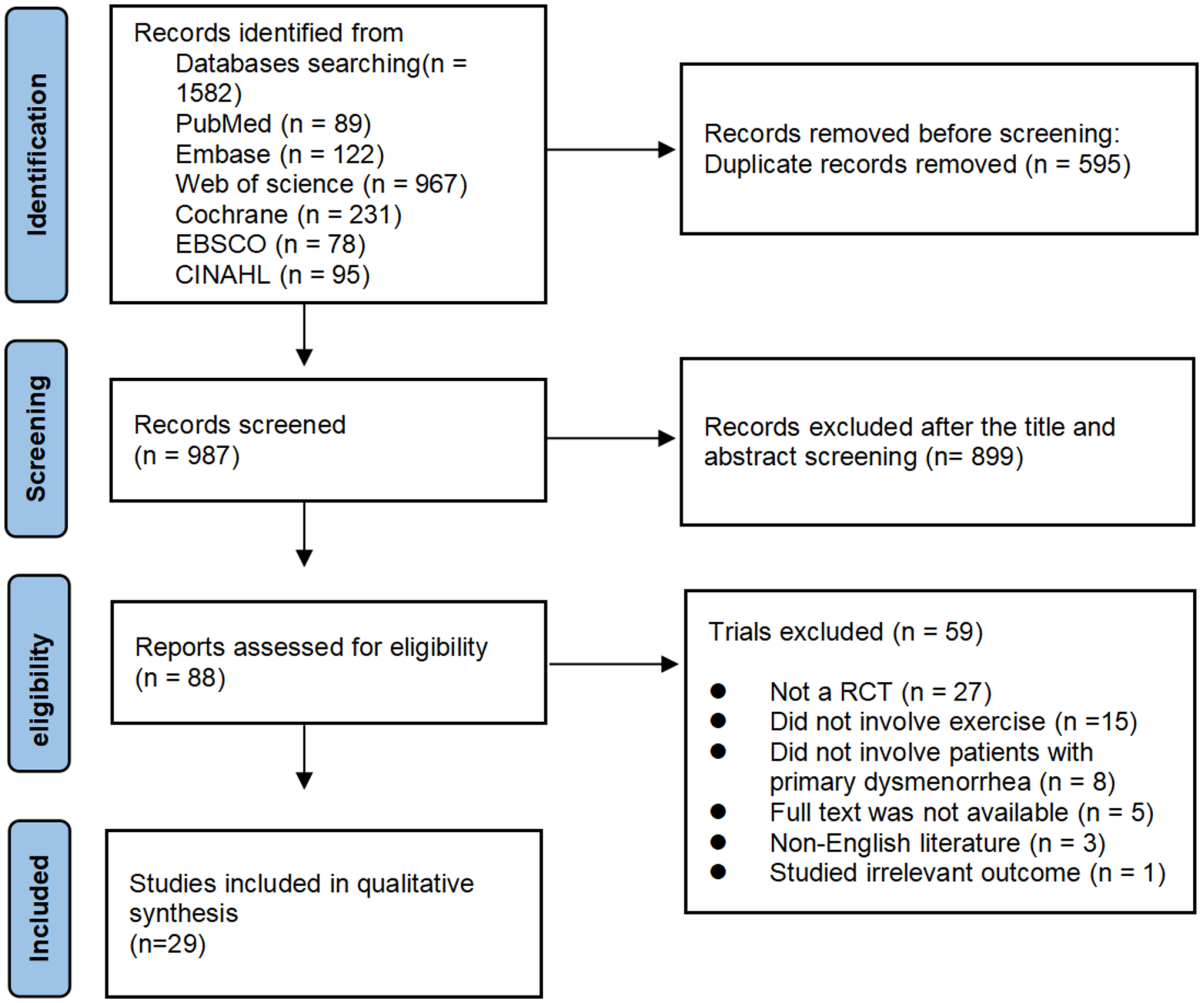
PRISMA flowchart of study selection.

As illustrated in Figure 1, a thorough search of six databases revealed 1,582 relevant papers. After removing duplicates, 987 papers were examined by reading titles and abstracts, resulting in the exclusion of 899 studies. After a thorough review of the full texts, 59 papers were excluded for the following reasons (1): not an RCT (*n* = 27) (2); no exercise (*n* = 15) (3); no patients with primary dysmenorrhea (*n* = 8) (4); full text not accessible (*n* = 5) (5); non-English literature (*n* = 3); and (6) study of an irrelevant outcome (*n* = 1). Finally, 29 papers matched the inclusion criteria (15–19, 24–47).

### 3.2 Characteristics of the included studies

The main characteristics of the interventions and the people involved are presented in Table 1. The studies included 1,268 individuals in 36 intervention groups and 927 in 29 control groups. In the study, each intervention group received one of the following exercise treatments: aerobic exercise, mind–body therapy, relaxation exercise, stretching exercise, strength training, or multicomponent training. Multicomponent training refers to any motion that includes two or more of the above workout aspects. The intervention’s duration ranged from 4 to 48 weeks. A total of 29 studies used the VAS as the primary outcome measure (15–19, 24–47). The age range of participants in the examined studies spanned from 14.57 to 37.4 years. A total of 36 intervention groups were categorized as follows: 5 groups participated in the aerobic exercise, 6 groups in mind–body therapy, 6 groups in relaxation exercises, 12 groups in stretching exercises, 5 groups in strength training, and 2 groups in multicomponent training. The number of weekly interventions varied from one to seven times per week, with an overall average of three times per week. Each session of the intervention lasted between 10 and 60 min on average. Finally, the time spent on the intervention varied between 15 and 315 min weekly, with an average of 89 min.

**Table 1:** Features of the research that made up this meta-analysis.

Study	Country	Age (years)	Participants in nodes	Intervention duration (week)	Frequency (times/week)	Minutes per session (min)	Outcome indicators
Elbandrawy & Elhakk (16)	Egypt	INT:22.40 ± 1.94 CON:22.47 ± 1.55	35 Aerobic exercise 35 Control	8	3	45	VAS
Elbandrawy & Elhakk (16)	Egypt	INT:22.37 ± 1.87 CON:22.47 ± 1.55	35 Strength training 35 Control	8	3	45	VAS
Samy et al. (45)	Egypt	INT:21.41 ± 1.49 CON:21.53 ± 1.47	49 Zumba exercise 49 Control	8	2	60	VAS
Arora et al. (17)	India	INT:20.4 ± 1.54 CON:20.73 ± 1.50	30 Aerobic exercise 30 Control	12	3–5	>50	VAS
Fathy et al. (41)	Egypt	18–22	50 Aerobic exercise 35 Control	12	3	30–40	VAS
Rezvani et al. (24)	Iran	INT:20.25 ± 2.02 CON:20.50 ± 1.79	20 Aquatic exercise 20 Control	12	3	60	VAS
Song & Kim (25)	Korea	INT:33.9 ± 3.5 CON:31.3 ± 4.5	15 Pilates 15 Control	12	2	50	VAS
Yang & Kim (26)	Korea	20–23	18 Yoga 18 Control	12	1	60	VAS
Rakhshaee (40)	Iran	INT(mean):20.86 CON(mean):20.45	50 Yoga 42 Control	8	7	20	VAS
Yonglitthipagon et al. (27)	Thailand	INT:19.71 ± 1.36 CON:20.6 ± 1.03	17 Mind–Body therapy 17 Control	12	2	30	VAS
Kirca & Celik (15)	Türkiye	INT:20.30 ± 0.46 CON:20.46 ± 0.50	30 Mind–Body Therapy 30 Control	12	1	60	VAS
Fallah & Mirfeizi (29)	Iran	INT:15.68 ± 0.95 CON:15.54 ± 0.94	19 Stretching exercise 19 Control	8	2	20	VAS
Fallah & Mirfeizi (29)	Iran	INT:15.53 ± 0.7 CON:15.54 ± 0.94	19 Relaxation exercise 19 Control	8	2	10	VAS
Fallah & Mirfeizi (29)	Iran	INT:15.76 ± 0.83 CON:15.54 ± 0.94	21 Multicomponent training 19 Control	8	2	20	VAS
Celik & Apay (19)	Türkiye	INT:20.14 ± 1.34 CON:20.21 ± 1.50	64 Relaxation exercise 60 Control	8	>3	30	VAS
Celenay et al. (37)	Türkiye	INT:20.0 ± 1.0 CON:20.0 ± 2.0	26 Relaxation exercise 29 Control	4	3	20–30	VAS
Azima et al. (38)	Iran	INT:21.4 ± 0.95 CON:21.08 ± 1.21	34 Relaxation exercise 34 Control	8	0.5	30	VAS
Azima et al. (38)	Iran	INT:20.73 ± 1.08 CON:21.08 ± 1.21	34 Strength training 34 Control	8	5	NA	VAS
Yildiz & Acaroglu (42)	Türkiye	INT:19.50 ± 1.41 CON:20.04 ± 2.01	50 Relaxation exercise 47 Control	12	7	45	VAS
Ozturk et al. (28)	Türkiye	INT:19.6 ± 1.2 CON:20.0 ± 1.1	22 Relaxation exercise 19 Control	8	7	10	VAS
Ozturk et al. (28)	Türkiye	INT:19.7 ± 1.8 CON:20.0 ± 1.1	22 Stretching exercise 19 Control	8	7	NA	VAS
Patel et al. (46)	India	INT(mean):21.35 CON(mean):21.28	60 Stretching exercise 60 Control	8	3	NA	VAS
Jaibunnisha et al. (43)	India	18–21	33 Stretching exercise 34 Control	8	6	10	VAS
Ibrahim et al. (30)	Saudi Arabia	INT:21 ± 1.34 CON:20.6 ± 1.29	11 Stretching exercise 11 Control	4	3	30–45	VAS
Ibrahim et al. (30)	Saudi Arabia	INT:21.2 ± 1.17 CON:20.6 ± 1.29	11 Stretching exercise 11 Control	4	3	30–45	VAS
Saleh & Mowafy (31)	Egypt	INT:20.52 ± 1.03 CON:21.06 ± 1.31	44 Stretching exercise 38 Control	8	4	20	VAS
Saleh & Mowafy (31)	Egypt	INT:20.6 2 ± 1.06 CON:21.06 ± 1.31	44 Strength training 38 Control	8	4	30	VAS
Shah et al. (32)	India	INT:20.8 ± 1.8 CON:20.8 ± 1.8	20 Stretching exercise 20 Control	8	4	20	VAS
Gamit et al. (33)	India	18–25	15 Stretching exercise 15 Control	4	6	NA	VAS
Shahr-jerdy et al. (34)	Türkiye	INT(mean):16 CON(mean):16	124 Stretching exercise 55 Control	8	3	20	VAS
Chen & Hu (35)	America	INT:21.25 ± 0.47 CON:21.26 ± 0.48	63 Stretching exercise 64 Control	48	3	50	VAS
Abbaspour & Najjar (44)	Iran	INT:16.56 ± 1.12 CON:16.56 ± 1.12	97 Stretching exercise 45 Control	8	7	40	VAS
Azima et al. (38)	Iran	INT:21.08 ± 1.21 CON:20.73 ± 1.08	34 Strength training 34 Control	8	5	NA	VAS
ZAID et al. (18)	Malaysia	INT:22.58 ± 0.79 CON:22.58 ± 0.90	12 Strength training 12 Control	8	5	20	VAS
Abbas et al. (36)	Egypt	INT:24.33 ± 2.79 CON:24.67 ± 2.49	15 Multicomponent training 15 Control	4	3	60	VAS
Şaşmaz & Bayram (47)	Türkiye	INT:23.9 ± 2.3 CON:24.2 ± 2.9	25 Yoga 25 Control	8	2	50	VAS

INT, intervention group; CON, control group; NA, not available; VAS, visual analog scale.

### 3.3 Effects of exercise on VAS in patients with primary dysmenorrhea

**Figure 2:**
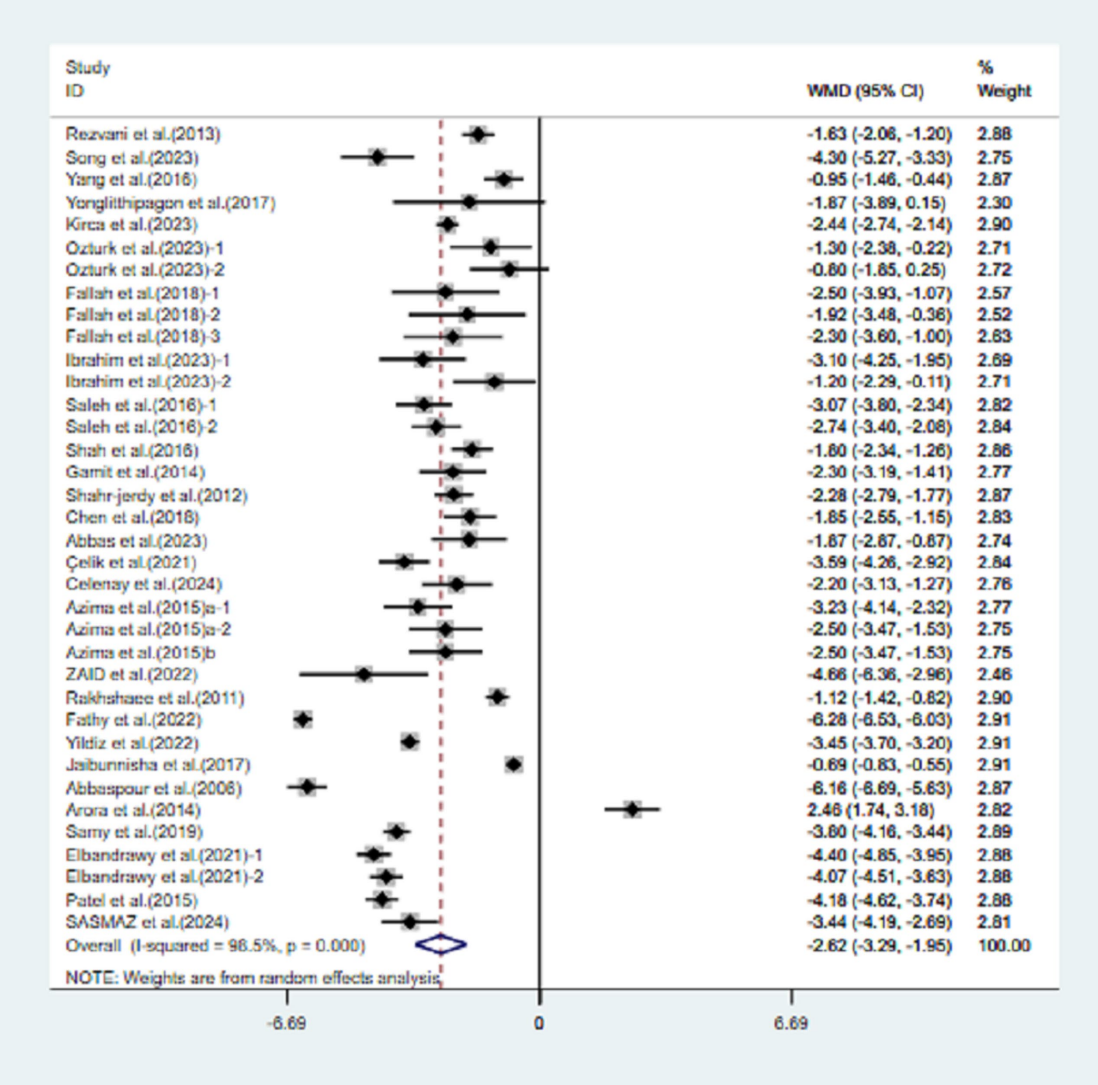
Meta-analysis results of the effects of exercise on the VAS in patients with primary dysmenorrhea.

A total of 29 studies reported VAS data. Compared to the control group, exercise decreased VAS scores in patients with primary dysmenorrhea (VAS, WMD = −2.62, 95% CI [−3.29, −1.95], *p* < 0.001), indicating that the pain level of the experimental group was significantly lower than that of the control group after intervention (Figure 2).

The meta-analysis demonstrated a significant degree of heterogeneity (98.5%) in the VAS. To address the variability present in the included studies and to identify modifiable elements of movement, additional meta-regression, subgroup analysis, and sensitivity analysis were conducted.

### 3.4 Sensitivity analysis

Sensitivity analysis revealed that the favorable impact of exercise on the VAS in patients with primary dysmenorrhea was steady and consistent in both direction and amplitude, even when individual studies were excluded (Supplementary Figure S1).

### 3.5 Risk of bias

**Figure 3:**
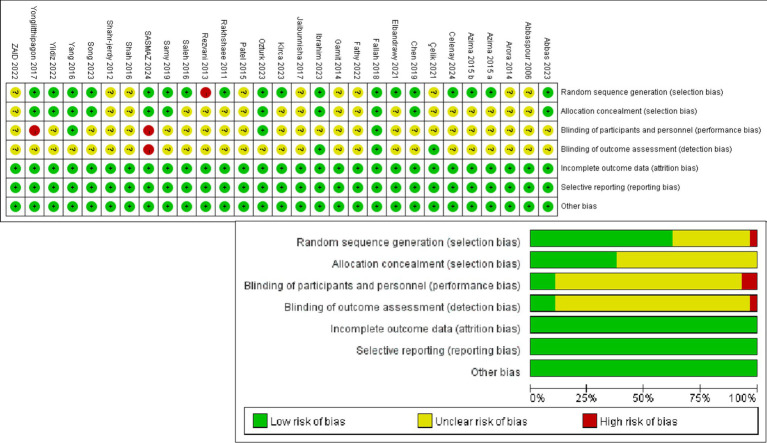
Cochrane risk of bias graph.

The Cochrane Handbook for Assessing Risk of Bias’s guiding principles were used to evaluate the caliber of the studies included (Figure 3). In terms of randomized sequences, one researcher (24) developed parity-based sequences deemed a substantial risk. Another 10 studies did not explain their technique of group assignment (17–19, 32–34, 41, 43, 44, 46); hence, these were classified as medium risk. In this situation, the other investigations were classified as low risk (15, 16, 25–31, 35–40, 42, 45, 47). Regarding allocation concealment, 18 studies did not give any information (15–19, 24, 31–34, 37–41, 43, 44, 46). These incidents were classified as intermediate risk. In this context, studies with allocation concealment were rated low risk (25–30, 35, 36, 42, 45, 47). Regarding research blinding, four studies reported single-masked procedures (19, 26, 28, 30), while one reported double-masked methods (29), all of which were considered low risk. Studies that lacked blinding of participants and outcome assessors were rated as high risk (27, 47), and the remaining studies were assessed as medium risk (15–18, 24, 25, 31–46). Concerning the integrity of the outcome data, all studies were classified as minimal risk, as none exhibited significant attrition during the intervention, which would have led to a high-risk assessment. Both groups received a low-risk assessment as the included studies did not indicate biased reporting of specific outcomes or other types of bias.

### 3.6 Publication bias

We used a funnel plot (Supplementary Figure S2) to investigate potential publication bias in greater depth. The discerned disparity suggests a tendency toward publishing bias. Egger’s test revealed no notable publication bias in VAS-related outcomes (*p* = 0.478 > 0.05) (Supplementary Figure S3).

### 3.7 Meta-regression

Meta-regression was used to obtain the center values of intervention length, frequency, session duration, and weekly time, and these values served as the critical points for subgroup analysis. The results showed that intervention duration, frequency, session duration, and weekly time significantly affected the VAS results. Regression analysis obtained the duration of the intervention (center value = 9.485714), frequency (center value = 3.421429), session duration (center value = 30.28571), and weekly time (central value = 91.07143) (Supplementary Figures S4, S5).

### 3.8 Subgroup analysis

**Figure 4:**
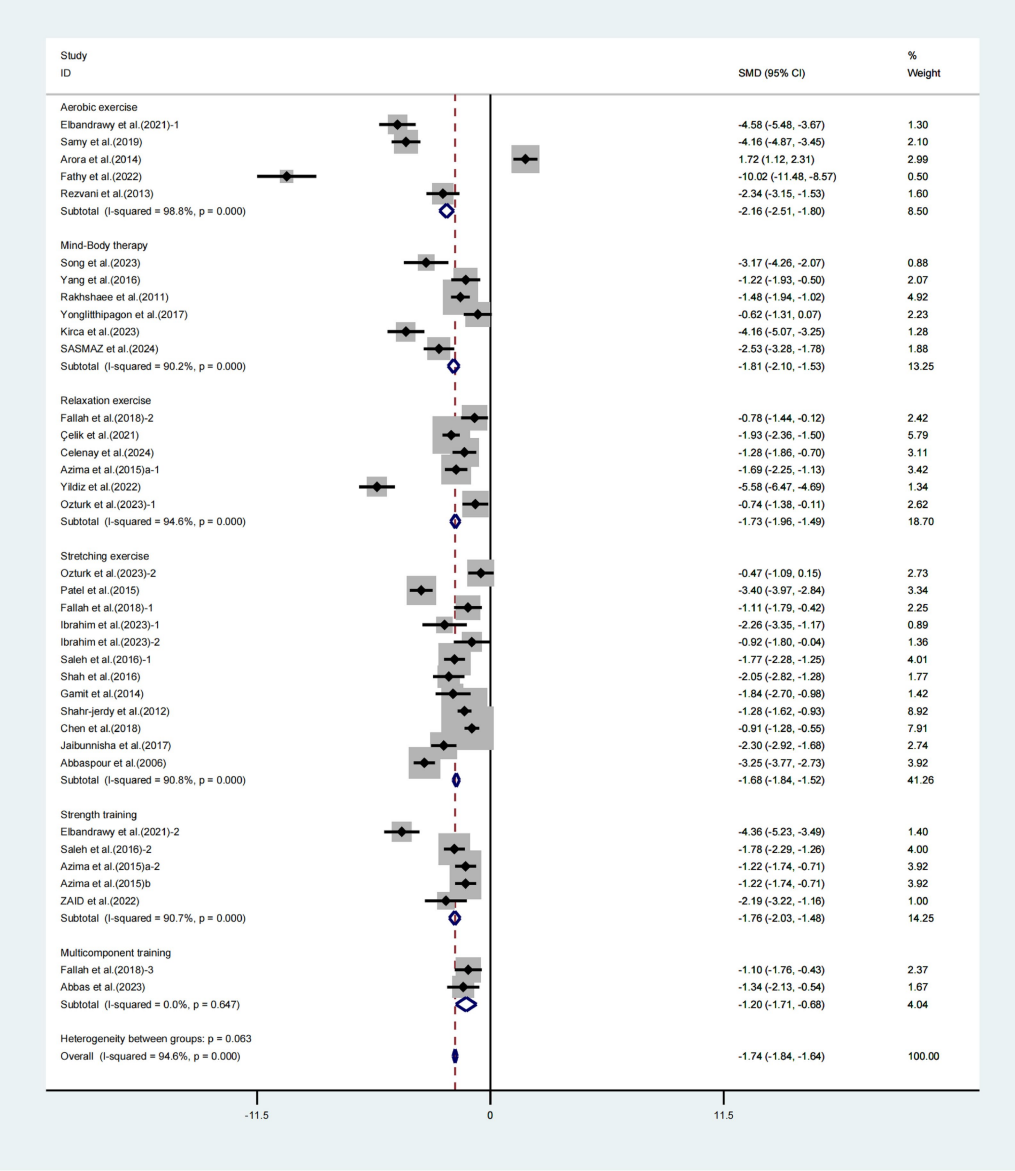
Meta-analysis results of the effects of different interventions on the VAS in patients with primary dysmenorrhea.

Stratified analysis by intervention type, aerobic exercise (WMD = −2.16, 95% CI [−2.51, −1.80], *I*^2^ = 98.8%, *p* < 0.001, Figure 4), mind–body therapy (WMD = −1.81, 95% CI [−2.10, −1.53], *I*^2^ = 90.2%, *p* < 0.001, Figure 4), relaxation exercise (WMD = −1.73, 95% CI [−1.96, −1.49], *I*^2^ = 94.6%, *p* < 0.001, Figure 4), stretching exercises (WMD = −1.68, 95% CI [−1.84, −1.52], *I*^2^ = 90.8%, *p* < 0.001, Figure 4), strength training (WMD = −1.76, 95% CI [−2.03, −1.48], *I*^2^ = 90.7%, *p* < 0.001, Figure 4), and multicomponent training (WMD = −1.20, 95% CI [−1.71, −0.68], *I*^2^ = 0.0%, *p* = 0.647, Figure 4) significantly improved VAS scores in patients with primary dysmenorrhea.

**Figure 5:**
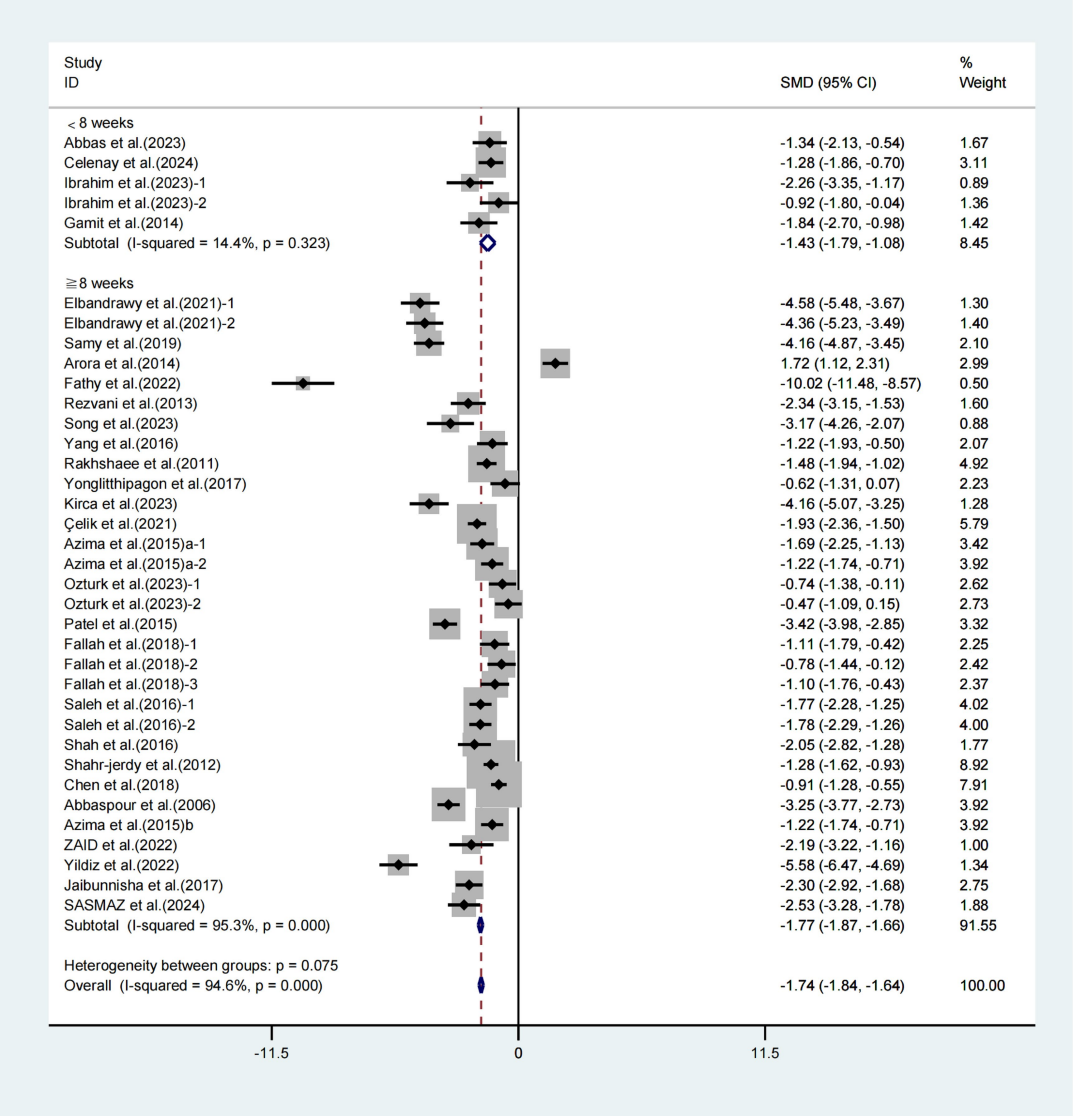
Meta-analysis results of the effect of intervention duration on the VAS in patients with primary dysmenorrhea.

Furthermore, subgroup analyses focusing on the duration of the intervention revealed notable enhancements in the VAS among patients experiencing primary dysmenorrhea who engaged in exercise for less than 8 weeks (WMD = −1.43, 95% CI [−1.79, −1.08], *I*^2^ = 14.4%, *p* = 0.323, Figure 5) and for 8 weeks or more (WMD = −1.77, 95% CI [−1.87, −1.66], *I*^2^ = 95.3%, *p* < 0.001, Figure 5). The exercise intervention lasting for 8 weeks or more demonstrated a notably greater impact on enhancing VAS scores in individuals with primary dysmenorrhea.

**Figure 6:**
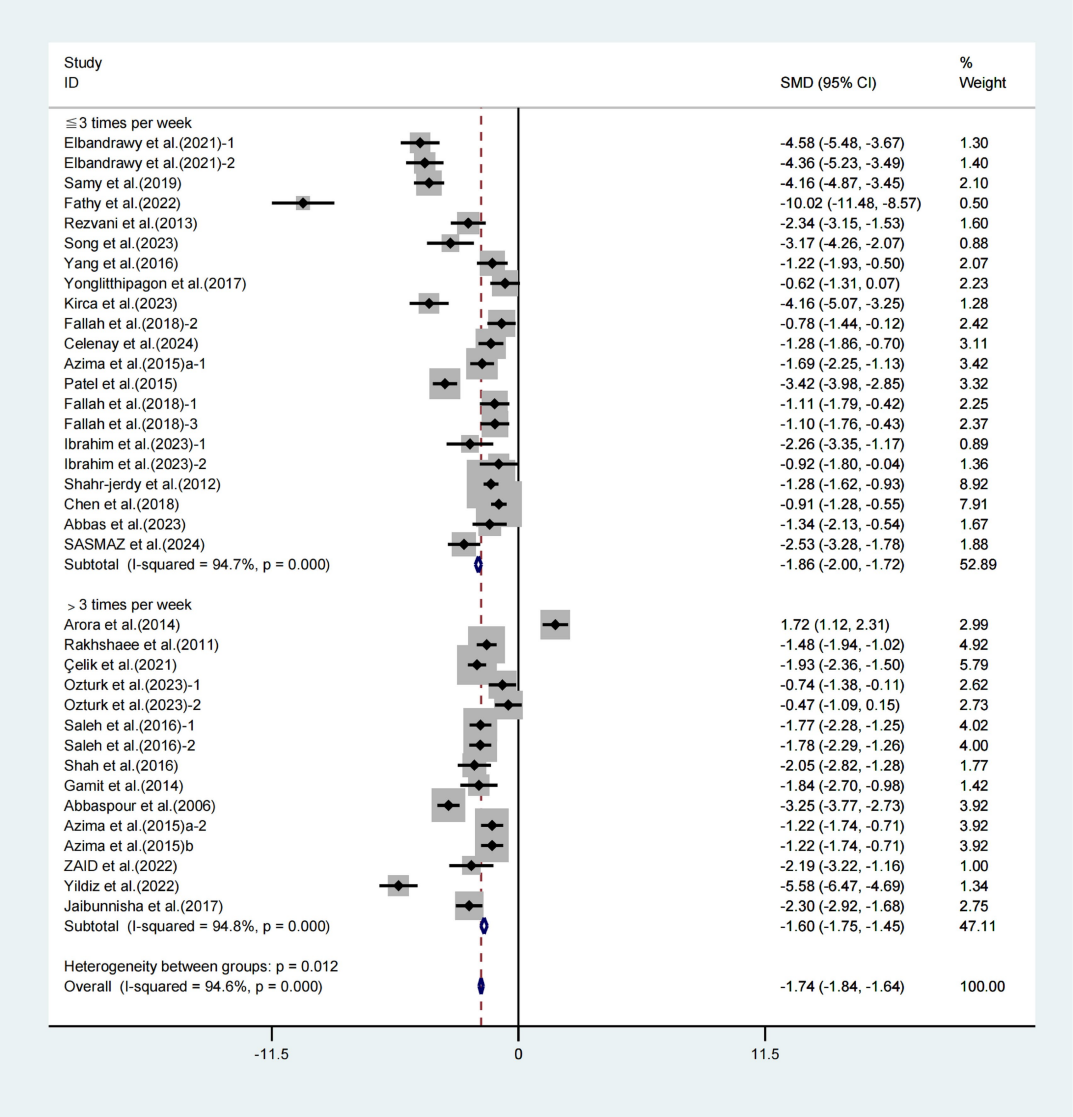
Meta-analysis results of the effect of frequency of intervention on the VAS in patients with primary dysmenorrhea.

Furthermore, when conducting subgroup analyses based on frequency, significant improvements in VAS scores were observed with weekly exercise intervention lasting ≤3 times (WMD = −1.86, 95% CI [−2.00, −1.72], *I*^2^ = 94.7%, *p* < 0.001, Figure 6) and weekly exercise intervention lasting >3 times (WMD = −1.60, 95% CI [−1.75, −1.45], *I*^2^ = 94.8%, *p* < 0.001, Figure 6). Specifically, the weekly exercise intervention lasting>3 times had a more significant effect on improving VAS scores in patients with primary dysmenorrhea.

**Figure 7:**
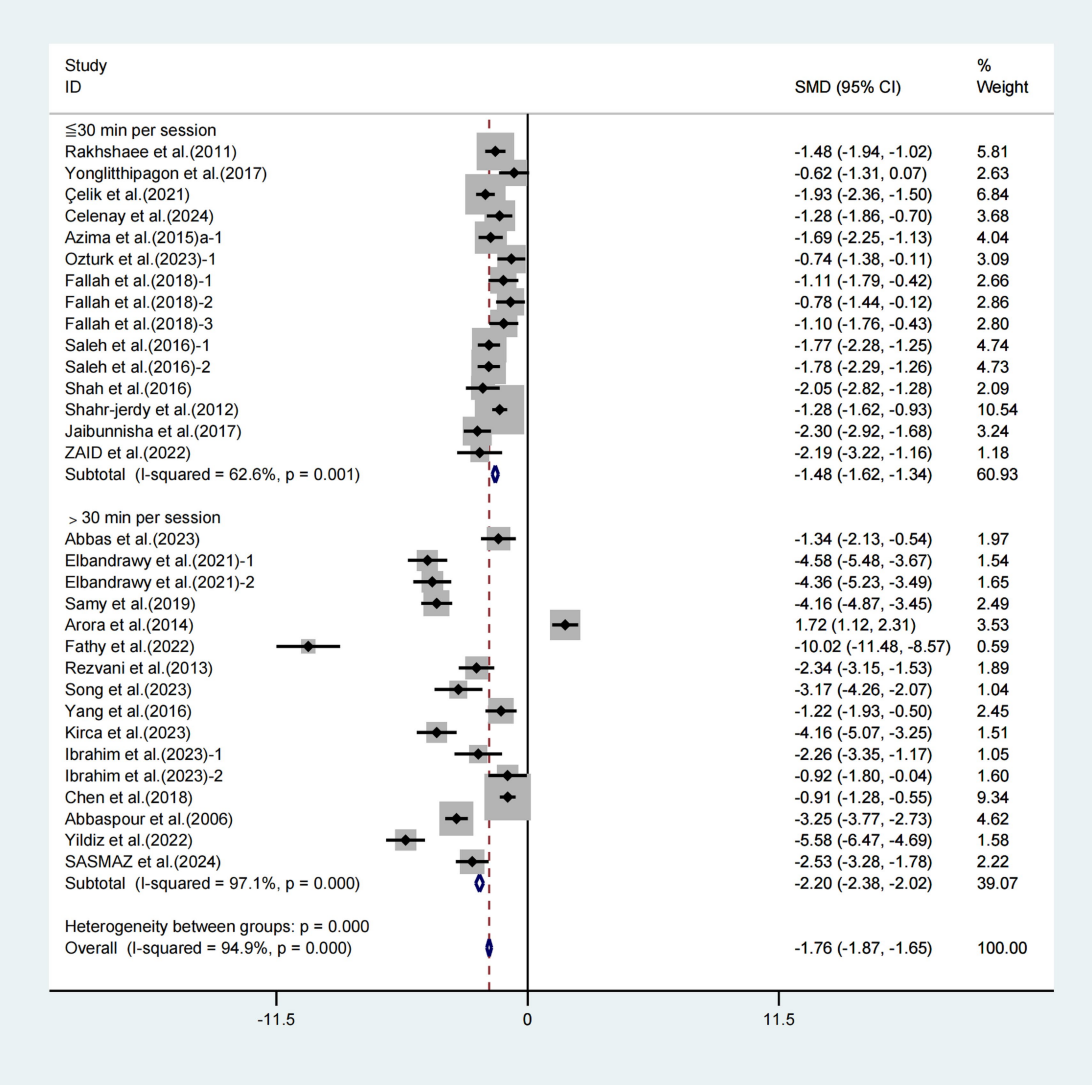
Meta-analysis results of the effect of duration of intervention per session on the VAS in patients with primary dysmenorrhea.

Moreover, when conducting subgroup analyses based on the duration of each session, significant improvements in VAS scores were observed with exercise intervention lasting ≤30 min (WMD = −1.48, 95% CI [−1.62, −1.34], *I*^2^ = 62.6%, *p* = 0.001, Figure 7) and exercise intervention lasting >30 min (WMD = −2.20, 95% CI [−2.38, −2.02], *I*^2^ = 97.1%, *p* < 0.001, Figure 7). Specifically, the exercise intervention lasting for >30 min had a greater effect on improving VAS scores in patients with primary dysmenorrhea.

**Figure 8:**
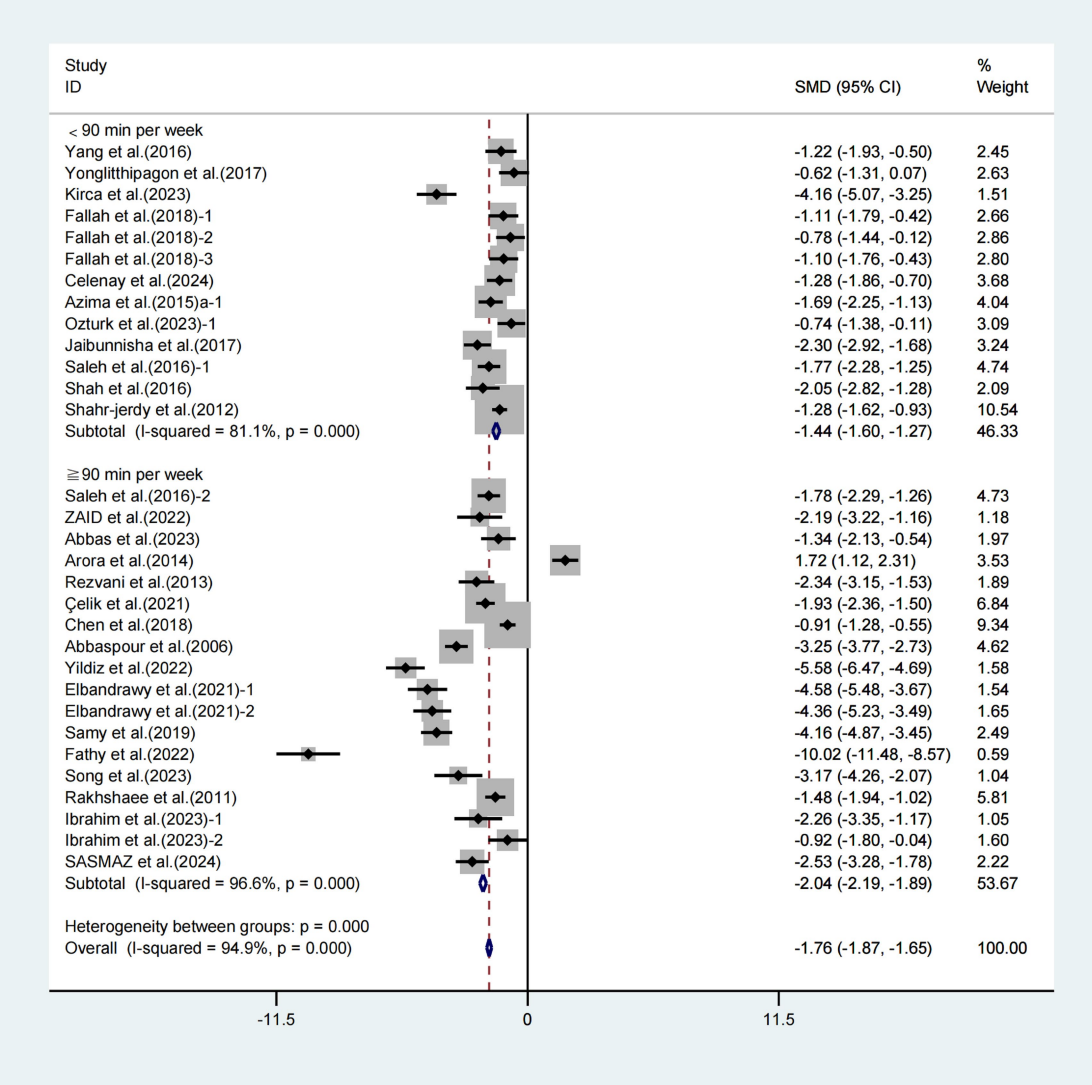
Meta-analysis results of the effect of duration of intervention per week on the VAS in patients with primary dysmenorrhea.

In the final analysis, subgroup analyses focused on weekly duration revealed noteworthy enhancements in VAS scores associated with exercise interventions lasting less than 90 min (WMD = −1.44, 95% CI [−1.60, −1.27], *I*^2^ = 81.1%, *p* < 0.001, Figure 8) and those lasting 90 min or more (WMD = −2.04, 95% CI [−2.19, −1.89], *I*^2^ = 96.6%, *p* < 0.001, Figure 8). Specifically, the weekly exercise intervention lasting ≥90 min had a greater effect on improving VAS scores in patients with primary dysmenorrhea.

### 3.9 SUCRA probability ranking

To evaluate and compare the efficacy of various exercise programs and control groups in enhancing VAS score in individuals with primary dysmenorrhea utilizing SUCRA: Strength training (71.0%) surpasses aerobic exercise (63.2%), followed by relaxation exercise (60.8%), stretching exercises (58.0%), multicomponent training (49.2%), and mind–body treatment (47.6%) (Supplementary Figure S6). The control groups accounted for 0.1%. The ranking indicated that strength training significantly enhanced VAS scores in patients with primary dysmenorrhea (Supplementary Table S1).

## 4 Discussion

### 4.1 Main findings

This study aimed to investigate the influence of exercise on pain intensity in adults with primary dysmenorrhea and formulate the most effective exercise prescription for these individuals. A total of 29 research studies showed that exercise might decrease discomfort (both severity and duration) in people with primary dysmenorrhea. Strength training and exercise for ≥8 weeks, >3 times per week for >30 min, and ≥ 90 min per week were shown to be more helpful in improving VAS scores.

### 4.2 Effect of exercise on pain relief in patients with primary dysmenorrhea

This thorough review and meta-analysis suggested that engaging in exercise may decrease VAS scores in patients with primary dysmenorrhea (VAS, WMD = −2.62, 95% CI [−3.29, −1.95], *p* < 0.001). Participants in the included trials usually exercised between day 3 and the completion of therapy or throughout the non-menstrual interval. The VAS is commonly used to assess the intensity of pain. Our meta-analysis showed that exercise decreased the 10-cm VAS score by approximately 2.62 cm. Matthewman et al. discovered that exercise intervention significantly reduced pain intensity in patients with primary dysmenorrhea, with an effect equivalent to a reduction of approximately 1.86 cm on a 10-cm VAS (48). Remedios et al. demonstrated that exercise resulted in a decrease of approximately 2.5 cm in the 10-cm VAS (49), which is similar to our findings. There are differences in the results of different studies. In general, exercise intervention can relieve the pain intensity of patients with primary dysmenorrhea. Furthermore, although exercise intervention has potential therapeutic benefits for patients with primary dysmenorrhea, whether the extent of improvement is clinically significant still needs further investigation and validation.

While earlier studies have not sufficiently elucidated the underlying causes and therapeutic processes through which exercise alleviates pain intensity in individuals experiencing primary dysmenorrhea, it achieves this through a range of beneficial effects. Exercise causes the release of *β*-endorphins at the cerebral, spinal, and peripheral levels (50). The body’s natural analgesics, β-endorphins, assist pain control (51). Exercise may effectively treat pain by boosting β-endorphin release, which inhibits pain pathways and may enhance the body’s pain threshold (18, 34). A study by Onan et al. revealed that a 6-week aerobic exercise program significantly reduced pain intensity in migraine patients and increased plasma β-endorphin levels, suggesting that aerobic exercise not only alleviates pain but also improves mood by elevating β-endorphin concentrations (52).

Moreover, exercise enhances anti-inflammatory cytokines and reduces pro-inflammatory cytokines. Consistent physical activity diminishes pro-inflammatory agents such as IL-6 and TNF-*α*, which activate damage receptors, while enhancing anti-inflammatory agents such as IL-10 (53–55). In addition, exercise induces muscle tissue to create regulatory macrophages, which effectively block the inflammatory response induced by pro-inflammatory cytokine-activated macrophages by secreting anti-inflammatory cytokines (56, 57). Kanna et al. (58) discovered that high-intensity aerobic exercise via an inflammatory cytokine-mediated mechanism reduced the discomfort associated with primary dysmenorrhea.

The endocrine components encompass prostaglandins, which govern the menstrual cycle and nociception. Physical activity may alter prostaglandin concentrations. Progesterone, a sex hormone, modulates the synthesis of prostaglandins, exhibiting an inverse link between the two (9, 59). Studies have revealed that high-intensity aerobic exercise enhances progesterone and reduces menstrual pain intensity compared to no exercise (58). Moreover, engaging in physical activity could alleviate dysmenorrhea symptoms by enhancing blood circulation and reducing tension in the uterine muscles. Indeed, core stabilization exercise therapies increase uterine circulation and minimize pelvic congestion (60, 61). Stretching has also been shown to promote circulation and muscular flexibility, which may help to alleviate stomach cramps (62).

It has been shown that exercise may not reduce the degree of discomfort in individuals with primary dysmenorrhea. Metheny and Smith discovered that exercise may worsen discomfort (63). Meanwhile, some studies have shown no clear link between physical exercise and dysmenorrhea (64–66). This discrepancy in data is attributable to the unique differences observed among patients. Every patient exhibited distinct physical attributes, varying levels of pain tolerance, and differing capacities for self-recovery, leading to a rich array of responses to therapeutic or exercise interventions. Furthermore, the degree of a patient’s dysmenorrhea may restrict their ability to participate in specific activities, reducing the efficacy of pain intensity alleviation. As a result, while designing a treatment plan for patients with primary dysmenorrhea, we suggest taking individual variations, general health state, and the quality and durability of the treatment program into full consideration. This tailored and thorough approach diminishes pain intensity while enhancing the overall quality of life for patients.

### 4.3 Subgroup analysis

This comprehensive review and meta-analysis have shown that exercise helps people with primary dysmenorrhea feel less pain. According to our analysis, VAS had a significant degree of heterogeneity (*I*^2^ = 98.5%). Heterogeneity in a meta-analysis refers to the variability in outcomes across different studies, which can arise from diverse factors such as study design, population characteristics, intervention measures, and measurement tools. To investigate the sources of heterogeneity, we conducted subgroup analysis, sensitivity analysis, and meta-regression. Our findings indicate that significant variations in exercise methods, intensity, frequency, and duration among the included studies may have contributed to the high heterogeneity observed in this meta-analysis (Supplementary Figure S5). Sensitivity analysis revealed that the favorable impact of exercise on VAS in patients with primary dysmenorrhea was steady and consistent in both direction and amplitude, even when individual studies were excluded (Supplementary Figure S1).

Various modalities of physical activity yield distinct outcomes in enhancing VAS scores among individuals experiencing primary dysmenorrhea. The investigation encompassed six distinct modalities of exercise intervention: aerobic exercise, mind–body therapy, relaxation techniques, stretching routines, strength training, and multicomponent training. According to the ranking probability analysis findings, strength training had the most significant ranking probability, followed by aerobic exercise and relaxation exercise. Therefore, strength training may be the most effective treatment option for patients with primary dysmenorrhea. This is comparable to the findings of Carroquino-Garcia et al.’s meta-analysis (48). Furthermore, various studies have proved the distinct advantages of strength training. Zaid et al. (18) discovered that isometric exercise might dramatically improve VAS scores after 8 weeks of intervention in individuals with primary dysmenorrhea. This conclusion is consistent with the findings of Azima et al. (39), who suggested isometric exercise to alleviate the harmful symptoms of dysmenorrhea. The majority of the studies on strength training interventions concentrated on the uterine region, emphasizing core training (31) and isometric training (16, 18, 38, 39). These uterine-specific exercise interventions facilitate the delivery of essential oxygen and nutrients to the uterine muscles and tissues, enhance blood circulation, and support repair processes. These also lower congestion and inflammation while optimizing the uterine microenvironment. This may provide a higher advantage than other therapies.

Our data indicate that an intervention lasting at least 8 weeks may improve VAS scores in patients with primary dysmenorrhea, outperforming interventions shorter than 8 weeks. In addition, this extended exercise intervention period showed the benefit of a longer duration in reducing patients’ pain intensity. These findings align with Rezvani et al.’s results (24), which showed that patients with primary dysmenorrhea experienced more excellent pain intensity relief after 8 weeks of aerobic exercise than after 4 weeks of training. In addition, after 4 and 8 weeks of non-stop intervention, a meta-analysis evaluated the impact of six different exercise modalities on reducing menstruation discomfort (5). This research found that all six activities decreased menstruation discomfort following an 8-week intervention. However, not all kinds of exercise impacted dysmenorrhea alleviation at the 4-week mark. Individuals with primary dysmenorrhea seeking better pain relief may pursue a suitable extension of the intervention duration to maximize its benefits.

In terms of intervention frequency, executing three or more treatments per week improved VAS scores in patients with primary dysmenorrhea. This is similar to earlier studies, likely due to the role of creating a regular exercise habit. It is noteworthy that a frequency exceeding three treatments per week demonstrated a more pronounced effect on enhancing VAS scores. Nevertheless, we did not overlook the potential benefit of administering fewer than three treatments per week, which may be contemplated in practice, contingent upon the duration of each session.

Our subgroup analysis revealed that therapies lasting up to 30 min, as well as those lasting more than 30 min per session, improved VAS scores in individuals with primary dysmenorrhea. Interventions lasting more than 30 min per session showed a more substantial effect on enhancing the VAS, consistent with previous research. However, some studies have demonstrated that excessively long exercise sessions not only fail to provide health advantages but may also harm the body (67). Nevertheless, the majority of intervention trials involving individuals with primary dysmenorrhea extended beyond 30 min, likely due to the reduced intensity, as longer treatments are necessary to ensure the effectiveness of the exercise. Ortiz et al. (68) found that frequent, appropriate-duration exercise had a favorable influence on the alleviation of dysmenorrheal symptoms. Short bursts of activity may not be enough to enhance function, and excessive exercise may worsen symptoms and impair the efficiency of the intervention. Future research should determine the precise impact of the time of each exercise session.

Nevertheless, our data suggest that merely considering frequency and session duration is insufficient to alleviate the influence of other variables. As a result, we integrated frequency and session duration to determine the weekly time allocated for each study project. The World Health Organization (WHO) recommends a minimum of 150 min per week of moderate-intensity aerobic physical activity or 75 min per week of vigorous-intensity aerobic physical activity (or a comparable combination) (69). Our subgroup analyses revealed that therapies lasting less than 90 min per week and over 90 min per week greatly improved VAS scores in patients with primary dysmenorrhea. The data suggest that interventions with a duration of at least 90 min per week significantly contributed to the improvement of the VAS scores.

As a result, a regimen that includes more than three treatments each week, each lasting at least 90 min, is more effective. It is recommended that persons with primary dysmenorrhea follow a specified exercise plan that gradually reduces the time of individual sessions to avoid negative consequences such as muscle injury or functional decline caused by decreased exercise tolerance. Increasing the weekly frequency of exercise may provide enough physical activity, which improves the treatment effect.

Based on our subgroup analyses of frequency, session duration, and timing within the week, we advocate that individuals experiencing primary dysmenorrhea strive for exercise sessions exceeding 30 min, emphasizing an increase in exercise frequency contingent upon a reduction in pain severity. However, past research has indicated that prolonged exercise duration may not provide health advantages and may have severe physical consequences. For example, Momma et al. (67) found that extensive training periods are one of the variables leading to severe dysmenorrhea in athletes. Exercise that is too brief fails to yield improvements, while excessive exercise may increase pain intensity in dysmenorrhea. This suggests that, for exercise therapies to effectively reduce pain intensity in individuals with primary dysmenorrhea, an adequate duration is required. As a result, we propose that single-session intervention time be limited while increasing exercise and increased frequency may be a preferable option.

### 4.4 Strengths and limitations of this study

The research we conducted is not without its limitations. To begin, the included studies focused on women with regular menstrual cycles, whereas those with irregular menstrual cycles were omitted; nonetheless, such women account for 5.0–35.6% of women of reproductive age (70). Second, our evaluation comprised 29 research studies, with 28 of them including individuals aged 14–28 and only one involving adults older than 28 years. This composition may restrict the scope of our results to include all women. In the statistical evaluation of the effect of exercise on pain intensity in patients with primary dysmenorrhea, the data were insufficient due to a lack of clear description of exercise frequency and single intervention time in some literature, which influenced the accuracy of the analysis results to some degree. Although this variable of pain duration was not fully explored in our study due to limited data from the included studies, future studies should investigate this variable to provide a more comprehensive assessment of treatment effects.

Moreover, our study focused exclusively on exercise interventions and did not account for the use of analgesic medications. Consequently, we were unable to assess the potential influence of such medications on the outcomes. Therefore, future studies should incorporate analgesic medications into their analyses to explore the combined effects of exercise and pharmacological interventions. Furthermore, the lack of consistency in exercise regimens throughout the included trials allowed for variances in intervention specifics, which contributed significantly to the variability. It is crucial to emphasize that although the included research used randomized controlled trials to implement exercise interventions, they needed to be more thorough. Consequently, subjective variables may have introduced a degree of bias into the quality rating process. Ultimately, given the evident variability in the results of the meta-analysis, it is imperative to exercise caution when interpreting the findings related to pain severity in individuals with primary dysmenorrhea.

## 5 Conclusion

In summary, physical activity decreases pain intensity in individuals with primary dysmenorrhea, with strength training potentially serving as the most efficacious intervention. This meta-analysis offers compelling evidence for clinicians to advise patients suffering from primary dysmenorrhea to engage in exercise more than three times weekly for durations exceeding 30 min, sustained over a minimum of 8 weeks, ultimately achieving a target of at least 90 min per week through increased exercise frequency.

## Data Availability

The original contributions presented in the study are included in the article/Supplementary material, further inquiries can be directed to the corresponding author.
