# Supplementary figures and images for: Efficacy and safety of therapeutic exercise for primary dysmenorrhea: a systematic review and meta-analysis
Source: Front Med (Lausanne). 2025 Feb 26;12:1540557. doi: 10.3389/fmed.2025.1540557 (PMC11896821; doi:10.3389/fmed.2025.1540557)

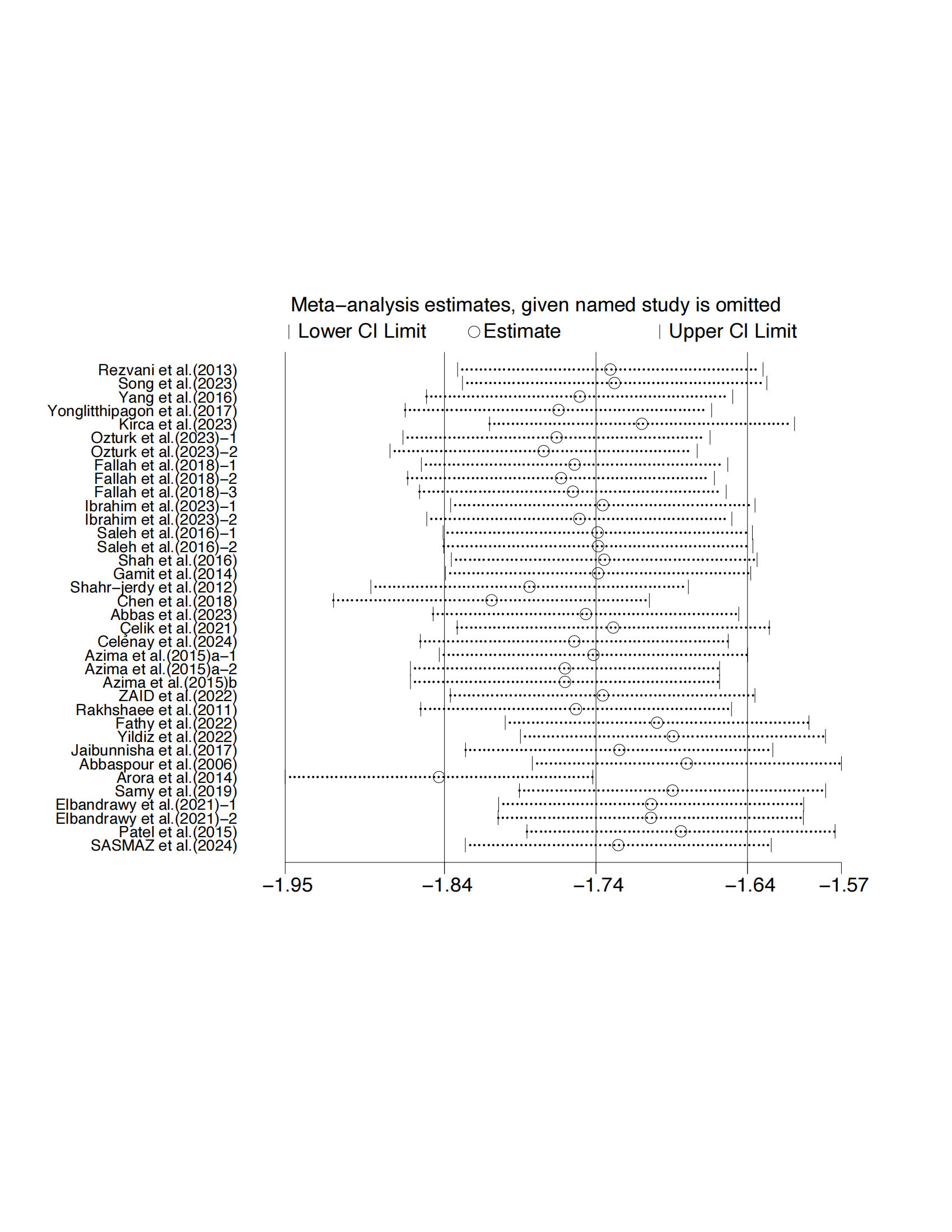

Supplement: Supplementary file 1 [file Image_1.JPEG]

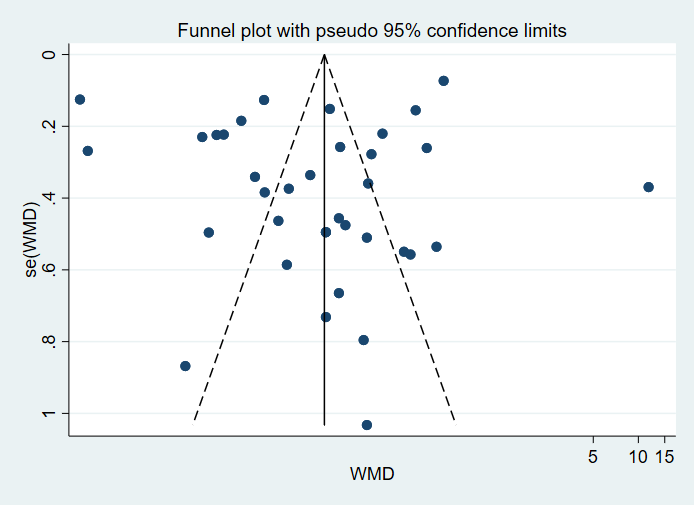

Supplement: Supplementary file 2 [file Image_2.JPEG]

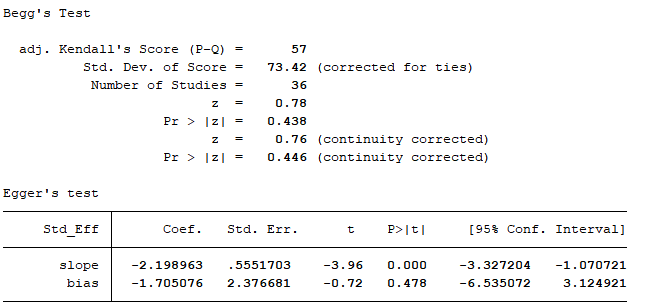

Supplement: Supplementary file 3 [file Image_3.JPEG]

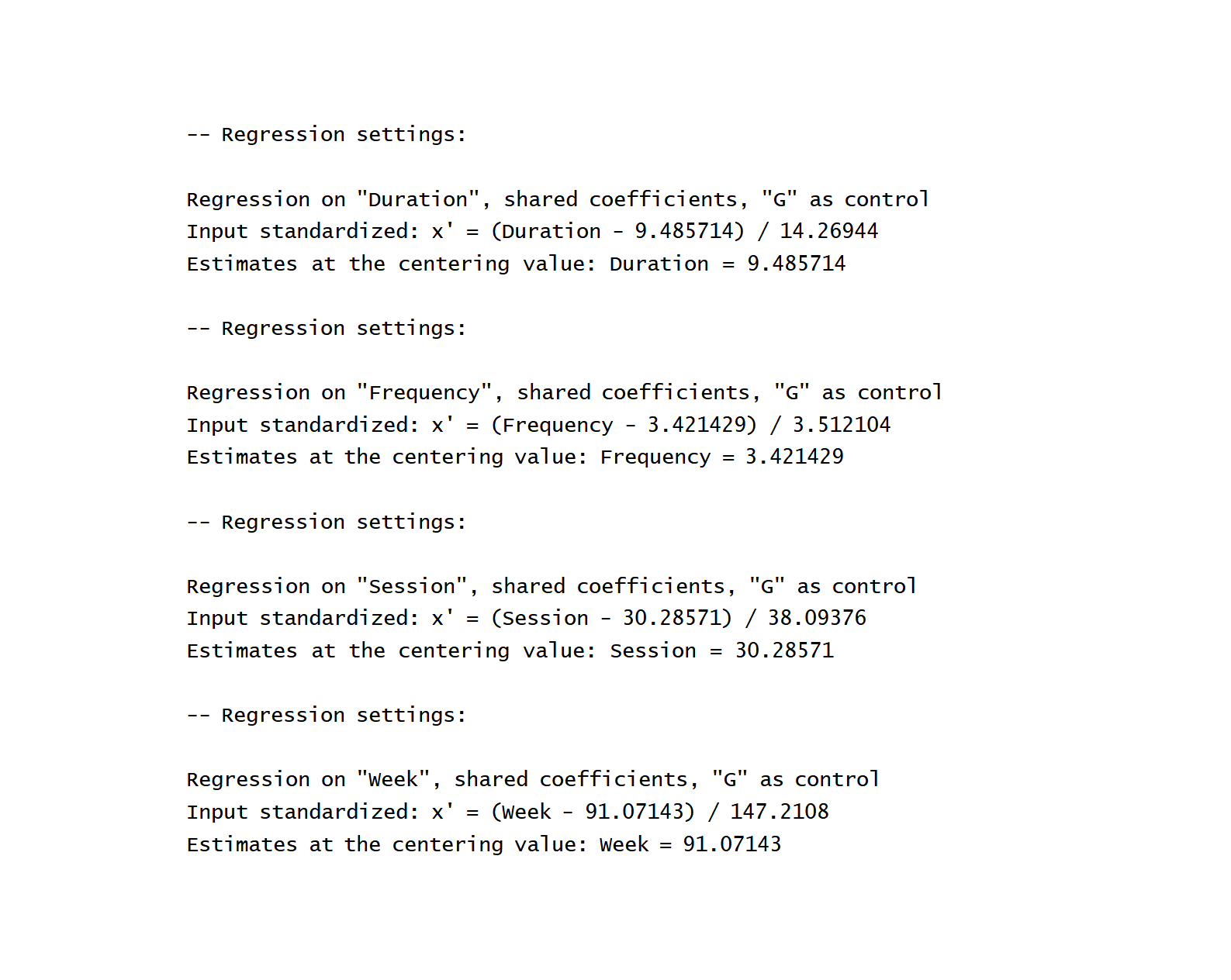

Supplement: Supplementary file 4 [file Image_4.JPEG]

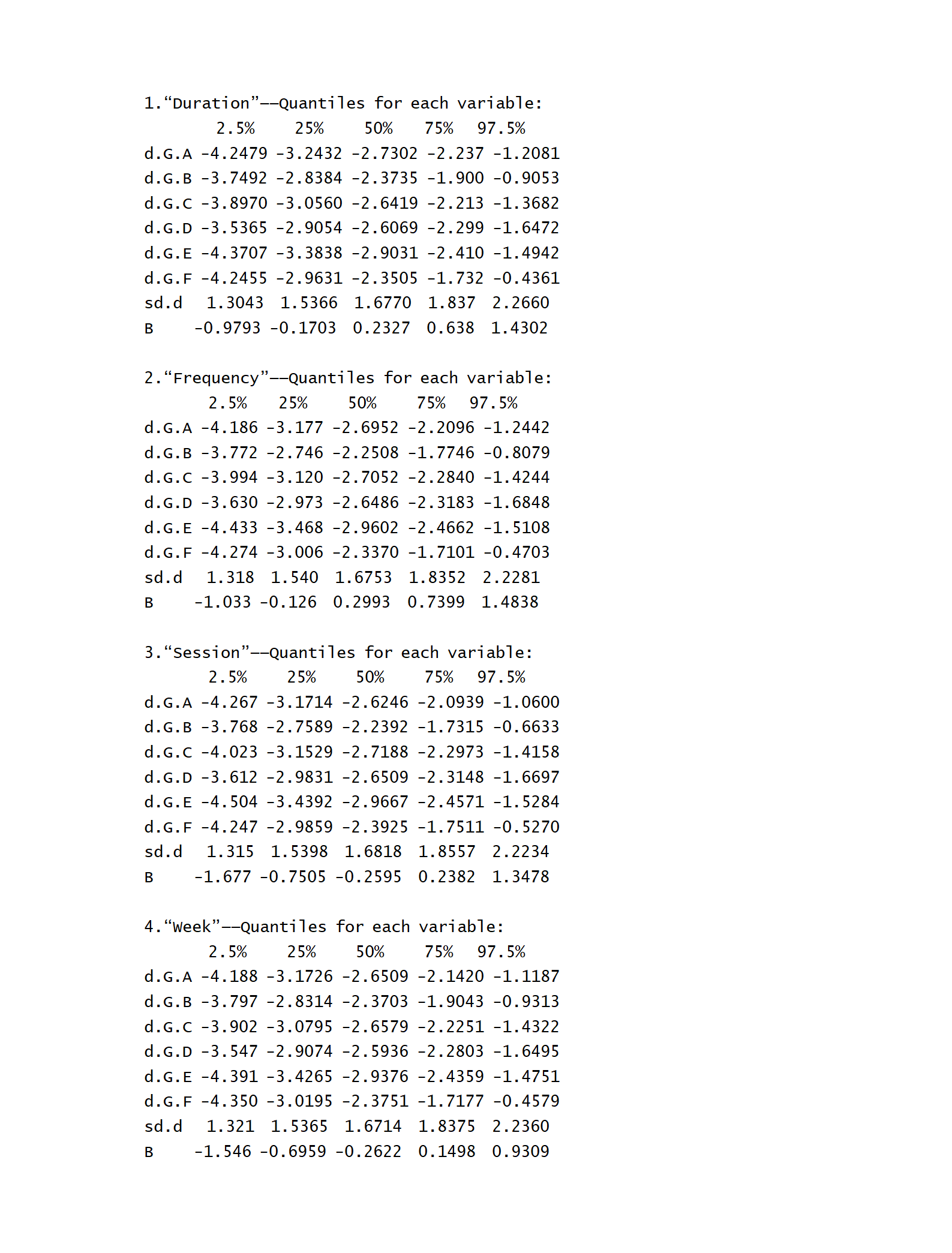

Supplement: Supplementary file 5 [file Image_5.JPEG]

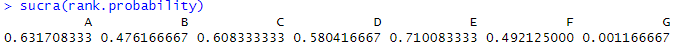

Supplement: Supplementary file 6 [file Image_6.JPEG]
