# Supplementary material for: Efficacy and safety of therapeutic exercise for primary dysmenorrhea: a systematic review and meta-analysis
Source: Front Med (Lausanne). 2025 Feb 26;12:1540557. doi: 10.3389/fmed.2025.1540557 (PMC11896821; doi:10.3389/fmed.2025.1540557)
Supplement: Supplementary file 7 [file Table_1.DOCX]

**Supplementary Table 1. Results of network meta-analysis among interventions and control group(WMD[95%CI])**

|  | **Aerobic Exercise** | **Mind-Body Therapy** | **Relaxation Exercise** | **Stretching Exercise** | **Strength Training** | **Multicomponent Training** | **No Intervention** |
| --- | --- | --- | --- | --- | --- | --- | --- |
| **Aerobic Exercise** | **Aerobic**  **Exercise** | 0.4 (-1.65, 2.5) | 0.07 (-1.77, 2.03) | 0.13 (-1.6, 1.82) | -0.18 (-2.12, 1.79) | 0.38 (-1.96, 2.74) | 2.73 (1.28, 4.24) |
| **Mind-Body Therapy** | -0.4 (-2.5, 1.65) | **Mind-Body**  **Therapy** | -0.34 (-2.17, 1.6) | -0.27 (-1.98, 1.49) | -0.57 (-2.6, 1.49) | -0.02 (-2.32, 2.29) | 2.34 (0.96, 3.81) |
| **Relaxation Exercise** | -0.07 (-2.03, 1.77) | 0.34 (-1.6, 2.17) | **Relaxation**  **Exercise** | 0.05 (-1.34, 1.48) | -0.26 (-2.05, 1.49) | 0.29 (-1.74, 2.28) | 2.67 (1.45, 3.93) |
| **Stretching Exercise** | -0.13 (-1.82, 1.6) | 0.27 (-1.49, 1.98) | -0.05 (-1.48, 1.34) | **Stretching**  **Exercise** | -0.32 (-2.02, 1.37) | 0.25 (-1.73, 2.17) | 2.62 (1.71, 3.55) |
| **Strength Training** | 0.18 (-1.79, 2.12) | 0.57 (-1.49, 2.6) | 0.26 (-1.49, 2.05) | 0.32 (-1.37, 2.02) | **Strength**  **Training** | 0.58 (-1.76, 2.85) | 2.93 (1.49, 4.43) |
| **Multicomponent Training** | -0.38 (-2.74, 1.96) | 0.02 (-2.29, 2.32) | -0.29 (-2.28, 1.74) | -0.25 (-2.17, 1.73) | -0.58 (-2.85, 1.76) | **Multicomponent**  **Training** | 2.35 (0.63, 4.15) |
| **No Intervention** | -2.73 (-4.24, -1.28) | -2.34 (-3.81, -0.96) | -2.67 (-3.93, -1.45) | -2.62 (-3.55, -1.71) | -2.93 (-4.43, -1.49) | -2.35 (-4.15, -0.63) | **No**  **Intervention** |
